# METTL3-Modulated *circUHRF2* Promotes Colorectal Cancer Stemness and Metastasis through Increasing *DDX27* mRNA Stability by Recruiting IGF2BP1

**DOI:** 10.3390/cancers15123148

**Published:** 2023-06-11

**Authors:** Miao Chen, Buning Tian, Gui Hu, Yihang Guo

**Affiliations:** Department of Gastrointestinal Surgery, The Third XiangYa Hospital of Central South University, Changsha 410013, China

**Keywords:** colorectal cancer, stemness, tumor metastasis, *circUHRF2*, DDX27 signaling, N6-methyladenine (m^6^A) modification

## Abstract

**Simple Summary:**

Colorectal cancer (CRC) is the third leading cause of cancer deaths worldwide, and no cure exists for most patients at advanced stages with distant metastasis. *CircUHRF2* has been aberrantly expressed in CRC, but its role in CRC growth and metastasis remains largely unclear. This study demonstrated that *circUHRF2* was upregulated in CRC and correlated with poor prognostic outcomes in CRC patients. Methyltransferase-like 3 (METTL3) facilitated *circUHRF2* expression through N6-methyladenine modification (m^6^A) modification. *circUHRF2* or METTL3 silencing suppressed in vitro cell stemness, migration, and invasion; and in vivo tumor growth and liver metastasis. Furthermore, *circUHRF2* is bound with insulin-like growth factor 2 mRNA-binding protein 1 (IGF2BP1) and increases the stability and expression of DEAD-box helicase 27 (*DDX27*) mRNA. Anti-cancer effect of *circUHRF2* silencing was counteracted by DDX27 overexpression. Our identification of the oncogenic roles of *circUHRF2* and METTL3 in CRC progression as well as their regulatory function through the IGF2BP1-DDX27 axis, has broadened our current knowledge about CRC and may help the future development of more efficient treatments.

**Abstract:**

Increasing evidence has implicated that circular RNAs (circRNAs) exert important roles in colorectal cancer (CRC) occurrence and progression. However, the role of a novel circRNA, *circUHRF2*, remains unknown in CRC. Our work aimed at identifying the functional roles of *circUHRF2* in CRC and illustrating the potential mechanisms. As assessed by quantitative real-time PCR (qRT-PCR), *circUHRF2* and methyltransferase-like 3 (METTL3) were highly expressed in CRC specimens and cells. Sanger sequencing and RNase R assays were performed to verify the ring structure of *circUHRF2*. Notably, aberrantly increased expression of *circUHRF2* was positively correlated with poor prognosis of CRC patients. Functional experiments indicated that CRC stemness, migration, and epithelial-mesenchymal transition (EMT) were suppressed by the knockdown of *circUHRF2* or METTL3. Mechanistically, METTL3 enhanced *circUHRF2* expression through N6-methyladenine (m^6^A) modification. Rescue experiments showed that overexpression of *circUHRF2* reversed the repressive effect of METTL3 silencing on CRC progression. Moreover, *circUHRF2* inhibited the loss of DEAD-box helicase 27 (DDX27) protein via promoting the interaction between insulin-like growth factor 2 mRNA-binding protein 1 (IGF2BP1) and *DDX27* mRNA. DDX27 knockdown repressed CRC malignant properties, which was counteracted by *circUHRF2* overexpression. The in vivo assays in nude mice demonstrated that *circUHRF2* or METTL3 silencing exerted a suppressive effect on CRC growth and liver metastasis via repressing DDX27 protein expression. Taken together, METTL3-mediated m^6^A modification upregulated *circUHRF2* and subsequently inhibited loss of DDX27 protein via recruitment of IGF2BP1, which conferred CRC stemness and metastasis. These findings shed light on CRC pathogenesis and suggest *circUHRF2* as a novel target for CRC treatment.

## 1. Introduction

Colorectal cancer (CRC) is the most common gastrointestinal malignant tumor and is ranked as the third leading cause of cancer deaths worldwide [1]. Despite the improvement in available screening and interventional treatments, CRC remains an increasing health burden globally [2]. The overall poor outcome has been mainly attributed to distant metastasis and diagnosis at an advanced stage [3]. Cancer stem cells are known to drive tumorigenesis and metastasis and represent a promising intervention strategy for CRC [4]. Therefore, a profound understanding of the underlying molecular mechanism of metastasis and stemness is conducive to developing curative therapy for CRC.

Circular RNAs (circRNAs) are single-stranded and closed non-coding RNAs containing a distinct loop structure formed by covalently linked ends [5]. Dysregulated expression of circRNAs in CRC has been extensively studied in the past decade [6]. It has been documented that *circAGFG1* contributed to metastasis and stemness in CRC by regulating YY1/CTNNB1 pathway [7]. A recent study by Yang et al. reported that *circUHRF2* (hsa_circ_0002359) was upregulated in CRC [8]; however, available evidence concerning the function of *circUHRF2* in the malignant properties of CRC cells remains limited.

N6-methyladenine modification (m^6^A) is the most abundant type of posttranslational modification in both mRNAs and non-coding RNAs [9]. M^6^A regulates gene expression by affecting various aspects of ribonucleic acid (RNA) metabolism, such as pre- messenger RNA (mRNA) processing, RNA nuclear export, RNA stability, and so on [10]. A recent study documented that fragile X messenger ribonucleoprotein 1 (FMR1) facilitated the tumorigenesis and metastasis of CRC cells by stabilizing epidermal growth factor receptor (*EGFR*) mRNA through m^6^A modification [11], suggesting the involvement of m^6^A-mediated regulation of RNA stability in the pathogenesis of CRC. The methylation of adenosine at the N-6 position is catalyzed by methyltransferase complexes known as “writers”, such as methyltransferase-like 3 (METTL3). Deletion of METTL3 led to reduced m^6^A level and was demonstrated to regulate tumor growth in glioblastoma [12], cervical cancer [13], and so on. Li et al. reported that METTL3 was highly expressed in CRC metastatic tissues, and its knockdown inhibited tumor progression through an m^6^A- SRY (sex determining region Y)-box 2 (SOX2)-insulin-like growth factor 2 mRNA-binding protein 2 (IGF2BP2)-dependent mechanism [14]. A recent study suggested that METTL3-mediated m^6^A of circ1662 facilitated metastasis of CRC via promoting yes-associated protein 1 (YAP1) nuclear transport [15]. Chen et al. reported that CRC liver metastasis was promoted by m^6^A modification on circNSUN2 [16]. So far, whether METTL3 affects CRC progression via m^6^A modification of *circUHRF2* has not been clarified.

Insulin-like growth factor 2 mRNA-binding protein 1 (IGF2BP1), a recently discovered “m^6^A-reader”, was reported to be overexpressed in CRC specimens [17]. Zhang et al. showed that IGF2BP1 inhibition exhibited antitumor roles in CRC via stabilization of LDHA [18]. Recently, circRNAs have been verified to modulate the expression of target genes via interaction with IGF2BP1. For instance, *circPTPRA* suppressed bladder cancer growth and metastasis via downregulation of m^6^A-modified MYC proto-oncogene, bHLH transcription factor (MYC) and fascin actin-bundling protein 1 (FSCN1) through interacting with IGF2BP1 [19]. In this context, we hypothesized that m^6^A-modified *circUHRF2* might affect CRC development by regulating DEAD-box helicase 27 (DDX27) protein expression via interaction with IGF2BP1.

In this work, we demonstrated that *circUHRF2* was highly expressed in CRC and correlated with a low survival rate. Knockdown of *circUHRF2* repressed CRC stemness, metastasis, and EMT process. Mechanistically, METTL3 enhanced *circUHRF2* expression via m^6^A modification, which restrained the loss of DDX27 protein via strengthening its interaction with IGF2BP1. These findings provide the first evidence for the therapeutic application of *circUHRF2* in CRC as a promising biomarker or treatment target.

## 2. Materials and Methods

### 2.1. Patients and Clinical Specimens

Sixty pairs of matched CRC primary tumor samples and adjacent nontumor tissues were collected from CRC patients who received surgical treatment at The Third XiangYa Hospital of Central South University. Tissues were rapidly frozen in liquid nitrogen and kept at −80 °C. Written informed consent was signed by all the participating patients. The Ethics Committee of the Third XiangYa Hospital of Central South University reviewed the ethics in the present study and approved our experimental procedures. We performed our research in accordance with the Declaration of Helsinki.

### 2.2. Cell Culture

Human colon epithelial cells line FHC and five CRC cell lines, including LoVo, SW480, SW620, HCT116, and HCT8, were obtained from the Cell Bank of Type Collection of Chinese Academy of Sciences (Shanghai, China). Cells were revived and cultured in RPMI-1640 media (Thermo Fisher, Waltham, MA, USA) supplemented with 10% fetal bovine serum (FBS, Thermo Fisher).

### 2.3. Cell Transfection or Infection

Short hairpin RNAs (shRNAs) against *circUHRF2*, METTL3, and DDX27 were provided by GenePharma (Shanghai, China). The sequences for shRNAs were shown as follows: sh-*circUHRF2*-1: 5′-GTATGATGATTGGAAAATGGA-3′; sh-*circUHRF2*-2: 5′-GATGATTGGAAAATGGATATA-3′; sh-METTL3-1: 5′-GCCTTAACATTGCCCACTGAT-3′; shMETTL3-2: 5′-GCAAGTATGTTCACTATGAAA-3′; sh-DDX27-1: 5′-GCAGAGGAAAGGTCTCAGTTT-3′; sh-DDX27-2: 5′-GCAGGAATTTGACTTGGCCTT-3′; sh- negative control (NC): 5′-TTCTCCGAACGTGTCACGT-3′. The full-length sequences of the *circUHRF2* gene (circBase ID: hsa_circ_0002359) were inserted into the pCD5-ciR vector (GENESEED, Guangzhou, China) to establish the *circUHRF2* over-expression plasmid. CRC cells were transfected with these segments using Lipofectamine 3000 (Thermo Fisher), according to the user’s guide. For transient transfection assay with shRNAs and plasmids, cells were harvested at 48 h for further experiments. For animal experiments, SW620 cells were stably infected with lentiviruses carrying sh-METTL3 or sh-*circUHRF2* (GenePharma) in the presence of 8 μg/mL polybrene (GenePharma). Stably transfected or infected cell clones were chosen by appropriate antibiotics (puromycin, 2–5 μg/mL, Sigma, Saint Louis, MO, USA) for at least one week after virus infection or plasmid transfection.

### 2.4. Subcellular Fractionation

The cells were resuspended in 500 µL ice-cold Native lysis Buffer (R0030, Solarbio, Beijing, China). The cells were homogenized by sonication using the Ultrasonic Cell Disruption System (Ymnl-450YC, YMNL Instrument Equipment Co., Ltd., Nanjing, China) at 20–25 KHz on ice. The cell lysate was centrifuged at 750× *g* for 10 min. The nuclear-containing pellet was washed with fractionation buffer and centrifuged at 1000× *g* for 10 min. The recovered supernatant from the previous step was centrifuged at 10,000× *g* for 10 min to sediment cell debris. The supernatant of the cytoplasmic fraction was transferred to a fresh tube. Both lysates were then subject to RNA isolation and qRT-PCR analysis. glyceraldehyde-3-phosphate dehydrogenase (GAPDH) and U6 small nuclear RNA (U6) served as the cytoplasmic and nuclear markers, respectively.

### 2.5. Sanger Sequencing and RNase R Treatment

The correctness of the back-slicing sites of *circUHRF2* was amplified using divergent primers and validated using Sanger sequencing. Briefly, total RNA was isolated from CRC cells with TRIzol reagent and reverse transcribed into complementary DNA (cDNA) with the TaqMan Reverse-Transcription Reagents (Applied Biosystems, Waltham, MA, USA), followed by an RNAse-H step (Ambion, Naugatuck, CT, USA). cDNA was amplified for 35 cycles using Phusion high-fidelity DNA polymerase (Thermo Fisher) in a total reaction volume of 25 μL, containing 400 nM of each primer and 160 µM dNTPs. The polymerase chain reaction (PCR) amplicons were purified from gel using the Qiaquick Gel Extraction Kit (Qiagen, Hilden, Germany) and subjected to Sanger sequencing by Sangon Biotech (Shanghai, China) on an ABI Hitachi 3730 sequencer.

For the RNase R digestion experiment, the total RNA of CRC cells was treated with 3U/μg RNase R for 1 h at 37 °C. Following treatment, quantitative Real-Time PCR (qRT-PCR) was adopted to assess *circUHRF2* and linear *UHRF2* levels.

### 2.6. Fluorescence In Situ Hybridization (FISH)

Subcellular localization of *circUHRF2* was examined by a FISH experiment. CRC cells were seeded on coverslips and incubated at 37 °C until 60% confluency. Next, cells were fixed with 4% paraformaldehyde (Sigma) and added with 0.3% Triton X-100 (Sigma) to induce complete membrane permeabilization. *CircUHRF2*-specific fluorescent probes were designed and synthesized by Sangon Biotech (Shanghai, China). The *circUHRF2* probe sequences were 5′-CY3-TTTACCATATCCAGTATGATGATTGGAAAATGGATATACCTTAT-3′. Hybridization procedures were conducted by incubating cells with the synthesized RNA probes in hybridization buffer (0.7 M NaCl, 0.1 M Tris pH 8.0, 0.1% SDS, 10 mM EDTA, and 1 mg/mL yeast transfer RNA) at 37 °C overnight. After annealing, the deoxyribonucleic acid (DNA) of chromosomes was counterstained using 50 µg/mL 1 4′,6-diamidino-2-phenylindole (DAPI, Solarbio). Subcellular distribution of *circUHRF2* was detected using Alexa Fluor 488 Signal-Amplification Kit (Thermo Fisher) and observed under a fluorescence microscope (Olympus, Tokyo, Japan).

### 2.7. Tumor Spheroid Formation

*CRC cells* were collected and seeded into a 6-well plate at a density of 100 cells per well. After two weeks of culture in RPMI-1640 media containing B27 (Thermo Fisher), 20 ng/mL epidermal growth factor (EGF, Sigma), 20 ng/mL basic fibroblast growth factor (bFGF, Thermo Fisher), and 4 μg/mL insulin (Sigma), the cell spheres were photographed under a light microscope (Zeiss, Oberkochen, Germany) and quantitatively analyzed.

### 2.8. Expression Profiling of CD133 by Flow Cytometry

Expression of the stem cell marker CD133 was confirmed by fluorescence-activated cell sorting (FACS). Briefly, CRC cells were harvested and resuspended in ice-cold PBS (Sigma). Alexa Fluor^®^ 488 labeled-CD133 primary antibody (1:2500, Abcam, Cambridge, UK, ab252126) was added to the cells and incubated for 1 h in the dark at room temperature. Cells were washed and immediately analyzed on a FACSymphony flow cytometer (BD Biosciences, San Jose, CA, USA).

### 2.9. Cell Invasion Assay

The invasive ability of *CRC cells was evaluated* using Transwell permeable inserts (8-μm, Corning, NY, USA). Transwell inserts were mounted to the multi-well plate, and the upper compartment was precoated with Matrigel (BD Biosciences). Fresh RPMI-1640 media containing 10% FBS was added to the lower compartment as a chemoattractant, and 5 × 10^4^ cells in 200 µL serum-free media were plated on the upper transwell inserts. After 24 h incubation, cells that invaded through the pores to the other side of membranes were fixed in 4% paraformaldehyde and stained with crystal violet (Beyotime, Haimen, China) for 10 min. The invaded cells were counted under a microscope.

### 2.10. Scratch Wound-Healing Assay

The confluent CRC cells were maintained in serum-free media. Then, a scratch was made using a pipette tip. After washing with PBS to remove the scratched cells, images were taken at 0 h and 24 h under a light microscope.

### 2.11. DDX27 mRNA Stability Assay

The stability of *DDX27* mRNA was tested using the transcription inhibitor actinomycin D (Thermo Fisher). CRC cells were incubated with actinomycin D at a final concentration of 5 µg/mL for 0, 2, 4, 8, and 12 h, respectively. The remaining expression level of *DDX27* mRNA was examined by qRT-PCR and normalized to its expression at 0 h.

### 2.12. Methylated RNA Immunoprecipitation (MeRIP) Assay

M^6^A enrichment was determined using the Magna MeRIP m6A Kit (Millipore, Billerica, MA, USA) following the manufacturer’s instructions. In brief, 18 μL of total RNA at a concentration of 1 μg/μL was mixed with 2 μL of Fragmentation Buffer 10× and heated at 94 °C for 5 min. After all the RNA was fragmented, size distribution was checked on 1.5% agarose gel. Magna ChIP Protein A/G Magnetic Beads (Millipore) were prewashed and incubated with anti-m^6^A antibody (Abcam, ab286164) or rabbit immunoglobulin G (IgG) for 30 min at room temperature. The immunoprecipitation mixture was prepared by incubating the beads above with fragmented RNA for 2 h at room temperature. The mixtures were placed on a magnetic separator, and methylated mRNAs were eluted using Elution Buffer, 2 μg of the RNA was served as the input, and the relative m^6^A-*circUHRF2* enrichment normalized to input was determined by qRT-PCR.

### 2.13. RNA Pull-Down Assay

The interaction between *circUHRF2* and IGF2BP1 was investigated by RNA pull-down assay using the Pierce Magnetic RNA-Protein Pull-Down Kit (Thermo Fisher). Briefly, CRC cells were lysed in ice-cold lysis buffer and centrifuged at 700× g. Total RNA was extracted and mixed with *circUHRF2*-specific probes. Cell lysate mixed with a random probe was used as a negative control. Subsequently, 50 µL Streptavidin Magnetic Beads were washed and incubated with the mixture above at room temperature for 30 min. The captured RNA-protein complexes were eluted and detected by Western blotting assay.

### 2.14. RNA-Protein Immunoprecipitation (RIP) Assay

RIP was performed using the EZMagna RIP Kit (Millipore, Billerica, MA, USA). The cell *lysate was prepared* using RNA lysis buffer. After centrifugation, the supernatant was incubated with magnetic beads coated with either an anti-IgG antibody (1:1000, ab172730, Abcam) or an anti-IGF2BP1 antibody (1:30, ab184305, Abcam). RIP lysate supernatant was used as input. Finally, the coprecipitated RNA was isolated, and the enrichment of *circUHRF2* and *DDX27* mRNA was determined by qRT-PCR experiment and normalized to the input.

### 2.15. Animal Experiments

BALB/C nude mice (four weeks old, male, *n* = 6 per group) were obtained from SJA Laboratory Animal Co., Ltd. (Changsha, China). To create a xenograft model, 1 × 10^7^ SW620 cells stably transfected shMETTL3 and shcircUHRF2 were subcutaneously injected into the nude mice. Tumor sizes were calculated by measuring the length and width (V = length × width^2^/2). Mice were euthanized four weeks later, and tumors were weighed. For in vivo liver metastasis assay, 1 × 10^6^ SW620 cells stably transfected shMETTL3 and shcircUHRF2 were injected into the distal tip of the spleen of mice according to previous studies [20,21]. Five weeks later, the mice were sacrificed to excise the liver tissues, and the visible metastatic tumor nodes were observed and counted. All animal studies were approved by The Third XiangYa Hospital of Central South University.

### 2.16. Hematoxylin and Eosin (H&E) Staining

Paraffin-embedded liver samples were sliced into 5-μm thicknesses and deparaffinized with xylene (Sigma). Slides were rehydrated with decreasing concentrations of ethanol solutions (100%, 95%, 80%, and 70%) and stained with hematoxylin (Solarbio) for 1 min. The slides were then stained with eosin (Solarbio) for 30 s and examined under the light microscope.

### 2.17. Immunohistochemistry (IHC)

The paraffin-embedded tumor samples were sliced to 5-μm thickness. After dewaxing, rehydration, and antigen retrieval, the sections were incubated with 3% bovine serum albumin (BSA, Sigma) for 1 h to block the nonspecific bindings. Primary antibodies against Ki-67 (1:200, Abcam, ab16667), CD133 (1:100, Abcam, ab284389) or DDX27 (1:2000, Thermo Fisher, A302-216A) was applied at 4 °C overnight. After washing, horseradish peroxidase (HRP)-conjugated secondary antibody (Abcam, ab6721) was added to the slides and incubated for 30 min. The stained samples were counterstained with DAPI and visualized under a fluorescence microscope.

### 2.18. RNA Isolation and Quantitative Real-Time PCR (qRT-PCR)

Total RNAs were extracted from CRC cells or tissue specimens using TRIzol reagent (Thermo Fisher). cDNA was synthesized using TaqMan Reverse-Transcription Reagents (Applied Biosystems, USA). qRT-PCR was conducted using SYBR Green Master Mix (Applied Biosystems), mixing 0.1 µL cDNA, 25 µL 2× SYBR Green Mix, and 400 nM of primers. The mixer was denatured at 95 °C for 3 min, followed by 35 cycles of 95 °C for 5 s and 60 °C for 10 s. Relative gene expression was calculated using the 2^−ΔΔCT^ method and normalized to GADPH expression. Primers used for the qRT-PCR were designed and synthesized by Sangon Biotech (Shanghai, China). Primer sequences are provided in Table 1.

### 2.19. Western Blot Analysis

Total protein was extracted from CRC cell or tissue samples using radio-immunoprecipitation assay (RIPA) buffer (Beyotime, Haimen, China) in the presence of a 1× protease inhibitor cocktail (Sigma). Protein concentration was determined by BCA Protein assay, and 30 μg of protein sample was separated on 10% sodium dodecyl sulfate polyacrylamide gel electrophoresis (SDS-PAGE). Proteins were transferred to a polyvinyl difluoride membrane. To block the nonspecific bindings, the membrane was incubated with 5% BSA for 1 h at room temperature. Primary antibodies against METTL3 (1:1000 dilution, Abcam, ab195352), DDX27 (1:3000 dilution, Abcam, ab177950, UK), E-cadherin (1 µg/mL, Abcam, ab231303), N-cadherin (1:10,000 dilution, Abcam, ab76011), Vimentin (1:3000 dilution, Abcam, ab92547), Slug (1:1000 dilution, Abcam, ab27568) or GAPDH (1:1000 dilution, Abcam, ab8245) was applied at 4 °C overnight. HRP-conjugated goat anti-rabbit secondary antibody was added to the membranes (1:3000 dilution, Abcam, ab205718) and incubated for 1 h. Protein signals were developed using the Amersham electrochemiluminescence (ECL) chemiluminescent detection system (GE Healthcare, South Plainfield, NJ, USA).

### 2.20. Statistical Analysis

SPSS Statistics 18.0 software (IBM Corporation, Armonk, NY, USA) was used to perform statistical analysis, and data are expressed as the mean ± standard deviation (SD). Kaplan–Meier method was used to calculate survival curves, and the significance was analyzed by log-rank test. The association between *circUHRF2* and the clinicopathologic parameters of the CRC patients was evaluated by a Chi-square test. Significant differences between the two groups were analyzed by *t*-test, and differences among multiple groups were analyzed using the one-way analysis of variance (ANOVA). According to a previous study [22], the median level of *circUHRF2*/METTL3 served as a cut-off value to divide the CRC patients into two groups (*n* = 30 per group): *circUHRF2*/METTL3 high expression and *circUHRF2*/METTL3 low expression groups. *p* < 0.05 was considered significant.

## 3. Results

### 3.1. CircUHRF2 Was Highly Expressed in CRC and Positively Correlated with Poor Prognosis

**Figure 1:**
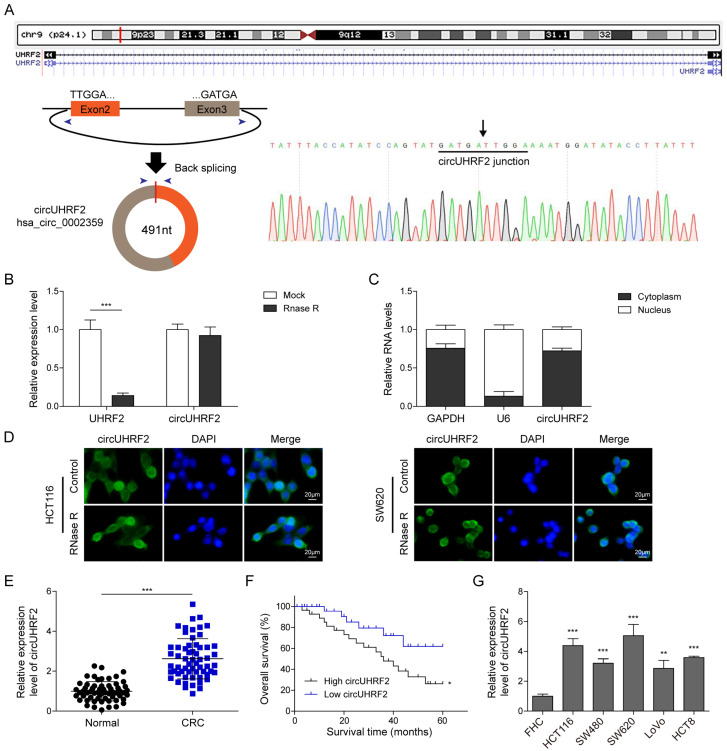
*CircUHRF2* was upregulated in colorectal cancer (CRC) and indicated a poor prognosis. (**A**) Molecular structure of *circUHRF2* shows that *circUHRF2* is a 491-bp gene originated from 2nd to 3rd exons in ubiquitin like with PHD and ring finger domains 2 (UHRF2). (**B**) The stability of *circUHRF2* in response to RNase R was examined by quantitative Real-Time PCR (qRT-PCR). (**C**) Cytoplasmic and nuclear fractions were separated, and relative expression of *circUHRF2* was detected by qRT-PCR. (**D**) Subcellular localization of *circUHRF2* was examined by a Fluorescence In Situ Hybridization (FISH) experiment. (**E**) *circUHRF2* expression in CRC samples and the normal controls were assessed by qRT-PCR. (**F**) Kaplan–Meier analysis of the correlation between *circUHRF2* expression and survival rate of CRC patients. (**G**) *CircUHRF2* expression in multiple CRC cells was determined by qRT-PCR. * *p* < 0.05, ** *p* < 0.01, and *** *p* < 0.001.

To verify the authenticity of *circUHRF2*, we performed Sanger-sequencing and identified that *circUHRF2* derives from the 2nd and 3rd exons of the *UHRF2* gene (Figure 1A). After RNase R treatment, linear UHRF2 was mostly digested, whereas *circUHRF2* remained unchanged, confirming the circular structure of *circUHRF2* (Figure 1B). After nucleus and cytoplasm fractionation, we detected that most of *circUHRF2* was located in the cytoplasm (Figure 1C). A FISH experiment further confirmed the cytoplasmic localization of *circUHRF2* in CRC cells treated with or without RNase R (Figure 1D). Next, we compared the relative expression of *circUHRF2* in CRC tissues (*n* = 60) and adjacent normal samples (*n* = 60) and observed that the *circUHRF2* level was significantly increased in CRC tissues (Figure 1E). Further, the upregulated expression of *circUHRF2* was correlated with a low survival rate (Figure 1F). A correlation analysis of the *circUHRF2* expression level and clinicopathological features of CRC patients indicated that patients with high *circUHRF2* expression exhibited larger tumor size and positively correlated with the tumor nodes metastasis (TNM) stage (Table 2). Consistently, CRC cell lines displayed enhanced expression of *circUHRF2* as compared to human colon epithelial cells FHC, and the highest expression was observed in HCT116 and SW620 cells (Figure 1G). Taken together, we have verified the circular structure of *circUHRF2* and identified its cytoplasmic localization in CRC cells. Upregulation of *circUHRF2* in CRC samples was statistically correlated with poor survival outcomes.

### 3.2. Knockdown of circUHRF2 Suppressed CRC Stemness, Migration, and EMT

**Figure 2:**
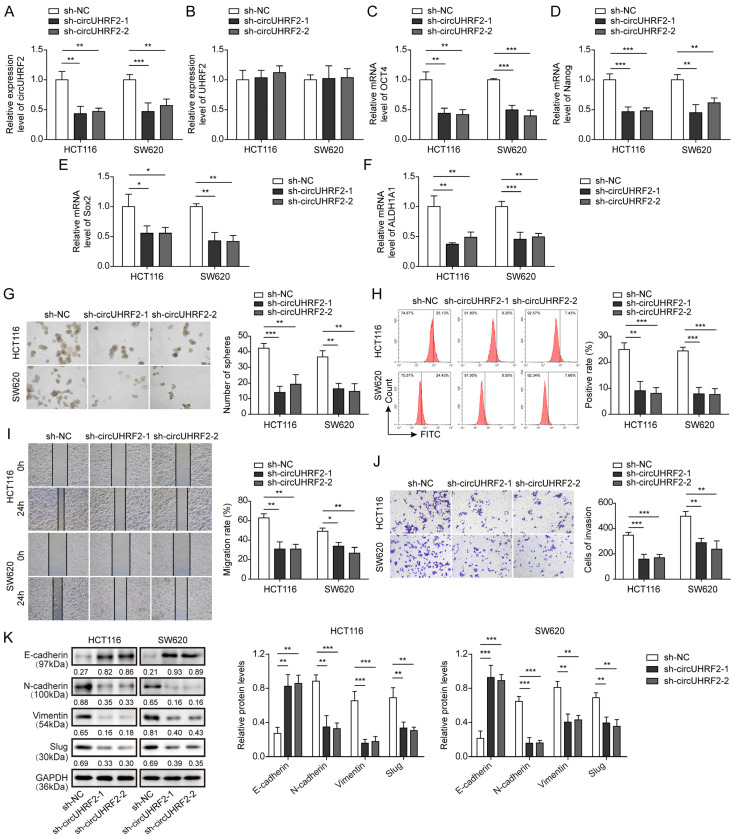
*CircUHRF2* knockdown suppressed colorectal cancer (CRC) stemness, migration as well as epithelial-mesenchymal transition (EMT). HCT116 and SW480 cells were transfected with sh*circUHRF2*. Relative expression of *circUHRF2* (**A**), linear ubiquitin like with PHD and ring finger domains 2 (UHRF2) (**B**), octamer-binding transcription factor 4 (OCT4) (**C**), Nanog (**D**), SRY-box transcription factor 2 (Sox2) (**E**), and aldehyde dehydrogenase 1 family member A1 (ALDH1A1) (**F**) in CRC cells was detected by qRT-PCR. (**G**) CRC cell sphere-forming ability was evaluated with and without *circUHRF2* knockdown. (**H**) Stemness marker CD133 was detected by flow cytometry. (**I**) A wound-healing experiment was performed to evaluate the migration ability. (**J**) Transwell assay analysis of invasion capacity. (**K**) Expression levels of EMT-related proteins, including E-cadherin, N-cadherin, Vimentin, and Slug, were measured by Western blotting, original blot see Supplementary File S1. * *p* < 0.05, ** *p* < 0.01, and *** *p* < 0.001.

To investigate the biological function of *circUHRF2* in CRC tumorigenesis and development, we silenced *circUHRF2* expression in HCT116 and SW620 cells (Figure 2A). Whereas the linear *UHRF2* level was not changed after transfection with sh*circUHRF2* (Figure 2B). qRT-PCR analysis showed that expression of stemness markers, including *OCT4* (Figure 2C), *Nanog* (Figure 2D), *Sox2* (Figure 2E) and *ALDH1A1* (Figure 2F), was largely reduced by *circUHRF2* knockdown. Notably, sh*circUHRF2* transfection inhibited the sphere-forming efficiency of CRC cells (Figure 2G). Flow cytometry showed that sh*circUHRF2* transfection remarkably decreased the percentage of CD133-positive cells (Figure 2H). Moreover, the knockdown of *circUHRF2* greatly attenuated CRC cell migration (Figure 2I) and invasion (Figure 2J) abilities. To address the question of whether EMT in CRC progression was affected by *circUHRF2*, we examined expression levels of key proteins involved in EMT. A higher expression of E-cadherin was observed in sh*circUHRF2*-transfected cells, whereas the expression of N-cadherin, Vimentin, and Slug was reduced (Figure 2K), suggesting that the EMT process was retarded by *circUHRF2* knockdown. These results above demonstrated that *circUHRF2* knockdown suppressed the malignant tumor properties of CRC cells, including tumorigenesis, stemness, migration, invasiveness, and EMT progression.

### 3.3. METTL3 Was Highly Expressed in CRC and Enhanced circUHRF2 Expression through m^6^A Modification

**Figure 3:**
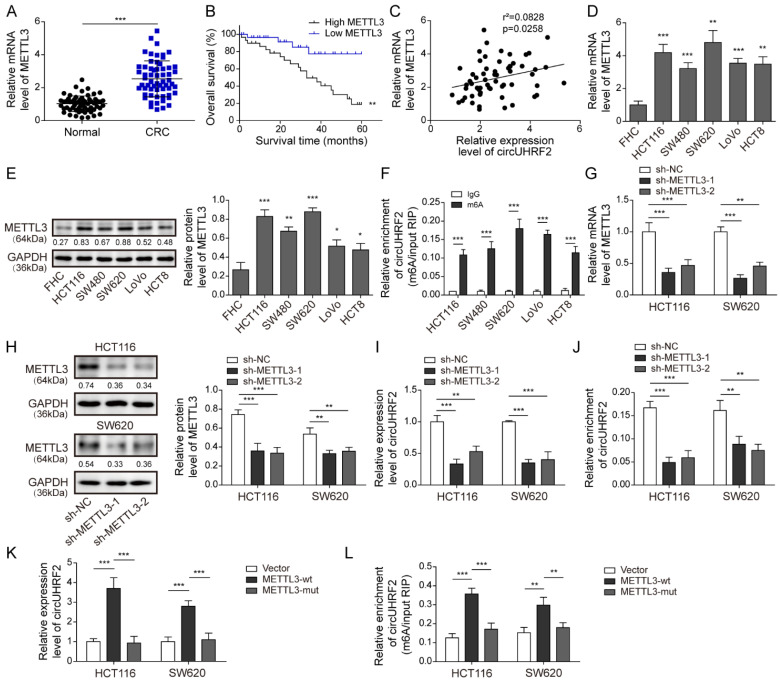
Upregulation of methyltransferase-like 3 (METTL3) enhanced *circUHRF2* expression through N6-methyladenine modification (m^6^A) modification in colorectal cancer (CRC) cells. (**A**) Quantitative Real-Time PCR (qRT-PCR) was performed to examine the expression of METTL3 in CRC and normal samples. (**B**) Kaplan–Meier analysis of the correlation between expression of METTL3 and survival rate. (**C**) The correlation between METTL3 and *circUHRF2* expression was evaluated in CRC tissue samples. (**D**,**E**) The relative expression level of METTL3 in CRC cell lines was detected by qRT-PCR and Western blotting. (**F**) The m^6^A level of *circUHRF2* in multiple CRC cells was assessed by MeRIP-PCR. HCT116 and SW620 cells were transfected with shMETTL3, and transfection efficiency was determined by qRT-PCR (**G**) and Western blotting (**H**). (**I**) *circUHRF2* expression in CRC cells after METTL3 silencing was assessed by qRT-PCR. (**J**) The m^6^A level of *circUHRF2* was evaluated by methylated RNA Immunoprecipitation (MeRIP)-PCR. (**K**) *circUHRF2* expression in CRC cells after transfection with METTL3-wt or METTL3-mut was detected by qRT-PCR. (**L**) The m^6^A level of *circUHRF2* in CRC cells transfected with METTL3-wt or METTL3-mut was measured by MeRIP-PCR. * *p* < 0.05, ** *p* < 0.01, and *** *p* < 0.001. Original blot see Supplementary File S1.

In accordance with the previous study [12], we detected a high aberrant expression of the m^6^A methyltransferase METTL3 in CRC tissue samples (Figure 3A). The median level of METTL3 served as a cut-off value to divide the CRC patients into two groups (*n* = 30 per group): METTL3 high expression and METTL3 low expression groups. As illustrated in Kaplan–Meier curve, upregulated METTL3 expression was correlated with a low survival rate (Figure 3B). Moreover, a positive correlation between METTL3 and *circUHRF2* was observed in CRC specimens (Figure 3C). Next, we profiled METTL3 in five CRC cell lines and found that *METTL3* mRNA (Figure 3D) and protein levels (Figure 3E) were markedly higher in CRC cells. As shown in Figure 3F, the m^6^A-*circUHRF2* level was significantly increased in CRC cells, especially in SW620 and HCT116 cells. To explore the potential mechanism by which *circUHRF2* was regulated by METTL3, we transfected CRC cells with shMETTL3 to silence METTL3 expression at both mRNA (Figure 3G) and protein (Figure 3H) levels. qRT-PCR results showed that *circUHRF2* expression was decreased in METTL3-depleted cells (Figure 3I). Interestingly, the SRAMP database predicted a series of m6A modification sites on *circUHRF2* (Supplementary Figure S1). Moreover, the m^6^A level of *circUHRF2* was significantly reduced upon shMETTL3 transfection, as indicated by the MeRIP-PCR assay (Figure 3J). Furthermore, we found that the expression of *circUHRF2* was enhanced by METTL3-wild type (wt) transfection; however, METTL3-mutant (mut) transfection did not affect *circUHRF2* expression (Figure 3K). In addition, the m^6^A level of *circUHRF2* was raised by METTL3-wt, which was not changed after METTL3-mut transfection (Figure 3L). Collectively, our data suggested that METTL3 facilitated *circUHRF2* expression through m^6^A modification, and its upregulation in CRC samples was correlated with poor prognosis.

### 3.4. Downregulation of METTL3 Suppressed CRC Stemness, Migration, and EMT by Decreasing circUHRF2 Expression

**Figure 4:**
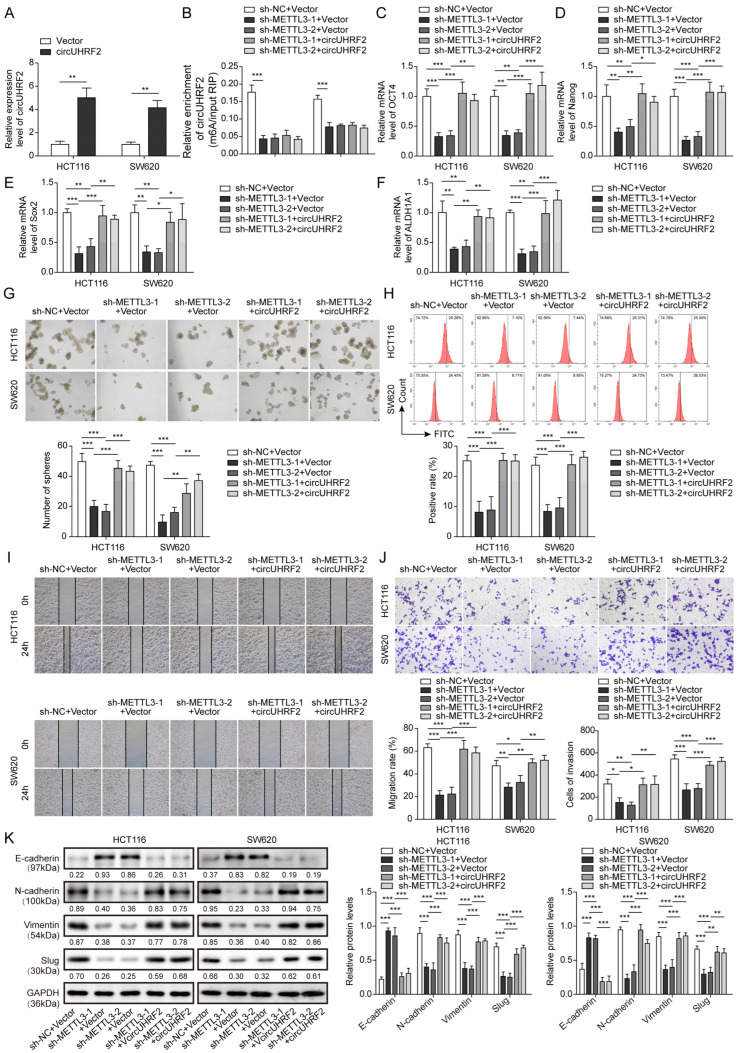
Colorectal cancer (CRC) stemness, migration, and epithelial-mesenchymal transition (EMT) were repressed by methyltransferase-like 3 (METTL3) depletion through reducing *circUHRF2* expression. HCT116 and SW480 cells were transfected with shMETTL3 together with or without *circUHRF2* overexpression plasmid. (**A**) Overexpression efficiency was evaluated by quantitative Real-Time PCR (qRT-PCR). (**B**) The N6-methyladenine modification (m^6^A) level of *circUHRF2* in CRC cells from different groups was evaluated by methylated RNA Immunoprecipitation (MeRIP)-PCR. Relative expression of the stemness markers, including octamer-binding transcription factor 4 (OCT4) (**C**), Nanog (**D**), SRY-box transcription factor 2 (Sox2) (**E**), and aldehyde dehydrogenase 1 family member A1 (ALDH1A1) (**F**), was determined by qRT-PCR. (**G**) CRC sphere-forming ability was measured. (**H**) Flow cytometry analysis of CD133 positive CRC cells. (**I**) A Wound-healing experiment was performed to evaluate the migration ability. (**J**) Transwell assay determined the invasive ability. (**K**) Expression levels of EMT-related proteins were assessed by Western blotting, original blot see Supplementary File S1. * *p* < 0.05, ** *p* < 0.01, and *** *p* < 0.001.

To reveal whether METTL3 affected CRC progression via modulating *circUHRF2*, we transfected CRC cells with shMETTL3 together with or without *circUHRF2* overexpression plasmid. The overexpression efficiency of *circUHRF2* was validated (Figure 4A). Knockdown of METTL3 remarkably reduced the m^6^A-*circUHRF2* level, and this change was not affected in shMETTL3-1/-2+*circUHRF2* groups (Figure 4B). As illustrated in qRT-PCR results, METTL3 knockdown markedly decreased the expression of stemness markers, including *OCT4, Nanog, Sox2*, and *ALDH1A1* (Figure 4C–F). In comparison, the expression of these molecules was rescued by *circUHRF2* overexpression (Figure 4B–E). In addition, CRC cell sphere formation was hindered (Figure 4G), and the number of CD133-positive cells was lowered by METTL3 knockdown (Figure 4H). However, *circUHRF2* overexpression abolished the inhibitory effect above (Figure 4G,H). As noted in Figure 4I,J, the silencing of METTL3 repressed migration and invasion ability, which was reversed by *circUHRF2* overexpression. Additionally, disrupted EMT progression by METTL3 knockdown was further recovered by *circUHRF2* overexpression (Figure 4K). These results revealed an efficient inhibition of METTL3 knockdown on the malignant properties of CRC cells, which was reversed by *circUHRF2* overexpression.

### 3.5. CircUHRF2 Directly Bound to IGF2BP1

**Figure 5:**
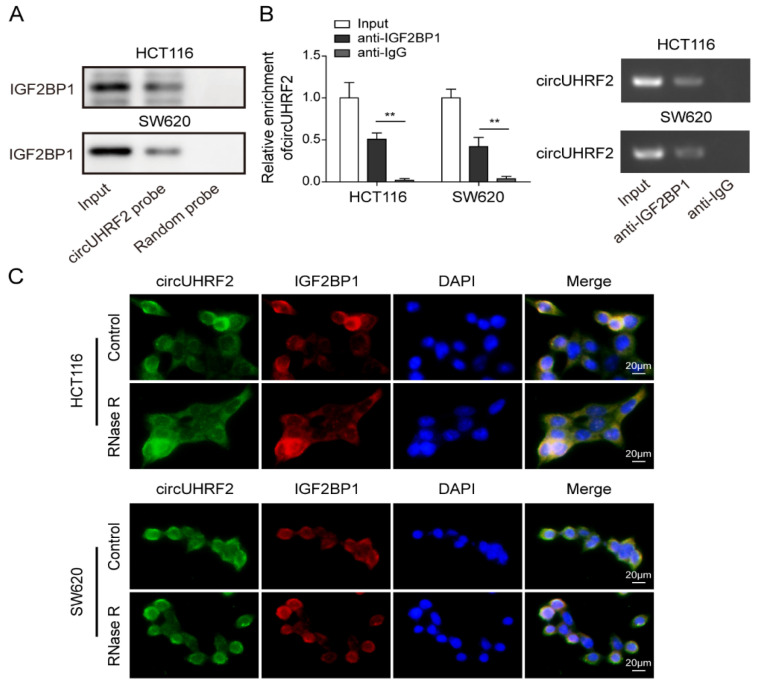
*circUHRF2* is directly bound to insulin-like growth factor 2 mRNA-binding protein 1 (IGF2BP1). (**A**) RNA pull-down assay analysis of the enrichment of IGF2BP1 by *circUHRF2* probe, original blot see Supplementary File S1. (**B**) The molecular association between *circUHRF2* and IGF2BP1 was validated by RNA-Protein Immunoprecipitation (RIP) assay. (**C**) The co-localization of *circUHRF2* and IGF2BP1 in CRC cells was observed by a Fluorescence In Situ Hybridization (FISH) experiment. ** *p* < 0.01.

To test whether IGF2BP1 was a binding partner of *circUHRF2*, we performed an RNA pull-down experiment and found that IGF2BP1 was remarkably enriched by a *circUHRF2*-specific probe (Figure 5A). The interaction between IGF2BP1 and *circUHRF2* was further verified by RIP assay (Figure 5B). Moreover, a FISH experiment revealed the cytoplasmic co-localization of *circUHRF2* and IGF2BP1 in both HCT116 and SW620 cells treated with or without RNase R (Figure 5C). The above findings confirmed our hypothesis that *circUHRF2* and IGF2BP1 formed an RNA-protein complex in the cytoplasm of CRC cells.

### 3.6. CircUHRF2 Restrained Loss of DDX27 Protein via Recruitment of IGF2BP1

**Figure 6:**
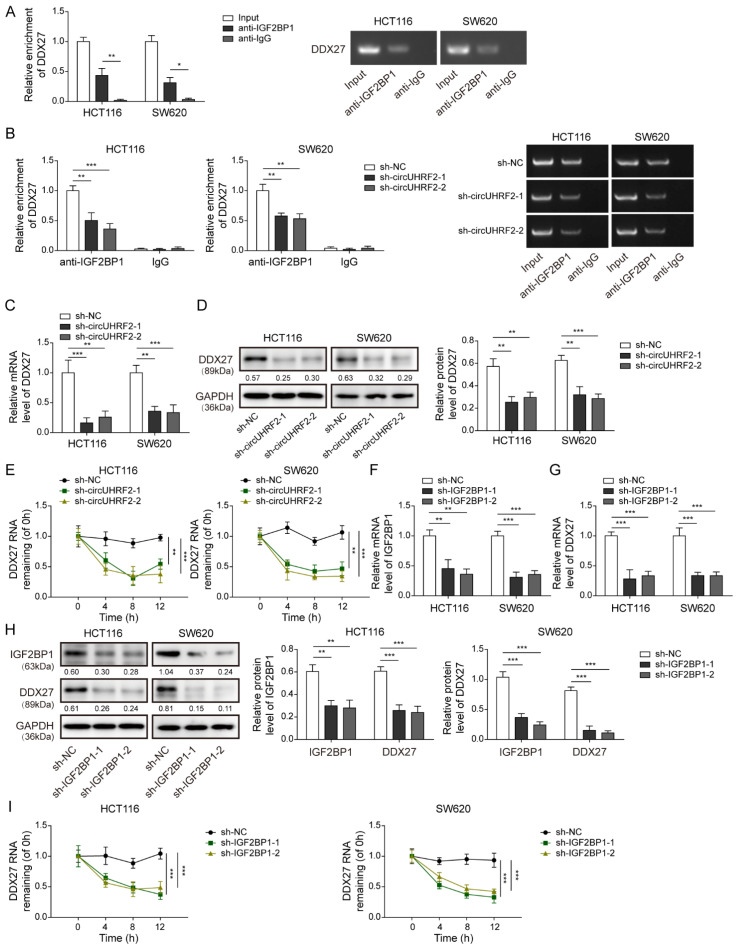
*CircUHRF2* recruited insulin-like growth factor 2 mRNA-binding protein 1 (IGF2BP1) to repress the loss of DEAD-box helicase 27 (DDX27) protein. (**A**) RNA-Protein Immunoprecipitation (RIP) assay was performed to determine the enrichment of *DDX27* mRNA by IGF2BP1 antibody. (**B**) Quantitative Real-Time PCR (qRT-PCR) detection of the enrichment of *DDX27* mRNA in the IGF2BP1 immunoprecipitate obtained by RIP and the result of agarose gel electrophoresis after qPCR. qRT-PCR (**C**) and Western blotting (**D**) measured *DDX27* mRNA and protein expression after *circUHRF2* knockdown. (**E**) After treatment with actinomycin D, qRT-PCR was performed to detect the remaining *DDX27* mRNA. qRT-PCR (**F**,**G**) and Western blotting (**H**) detected IGF2BP1 and *DDX27* mRNA and protein expression in shIGF2BP1-1/-2-transfected CRC cells. (**I**) The remaining *DDX27* mRNA after transfection with shIGF2BP1-1/-2 was assessed by qRT-PCR. * *p* < 0.05, ** *p* < 0.01, and *** *p* < 0.001. Original blot see Supplementary File S1.

As predicted by Cirinteractome and Starbase databases, there were putative binding sites between IGF2BP1 and *circUHRF2*/*DDX27* mRNA. To verify the predicted interaction between IGF2BP1 and *DDX27* mRNA, we performed a RIP assay and observed an increase in the enrichment of *DDX27* mRNA in complexes coprecipitated with IGF2BP1-specific antibody (Figure 6A). However, the enrichment of *DDX27* mRNA was notably reduced when the cells were transfected with sh*circUHRF2* (Figure 6B). In addition, DDX27 expression was inhibited by *circUHRF2* knockdown at both mRNA (Figure 6C) and protein (Figure 6D) levels. Moreover, the loss of *DDX27* mRNA in response to actinomycin D evidently declined after *circUHRF2* silencing (Figure 6E). Additionally, the mRNA and protein levels of DDX27 were reduced in IGF2BP1-depleted cells (Figure 6F–H). In Addition, the degradation of *DDX27* mRNA was promoted by IGF2BP1 silencing (Figure 6I). Our results provided the first experimental evidence for the direct interaction between IGF2BP1 and *DDX27* mRNA. Importantly, we showed that *circUHRF2* knockdown disrupted IGF2BP1-DDX27 complex formation to inhibit endogenous expression of *DDX27* mRNA and result in loss of DDX27 protein.

### 3.7. CircUHRF2 Silencing-Mediated Inhibition in CRC Stemness, Migration, and EMT Was Reversed by DDX27 Overexpression

**Figure 7:**
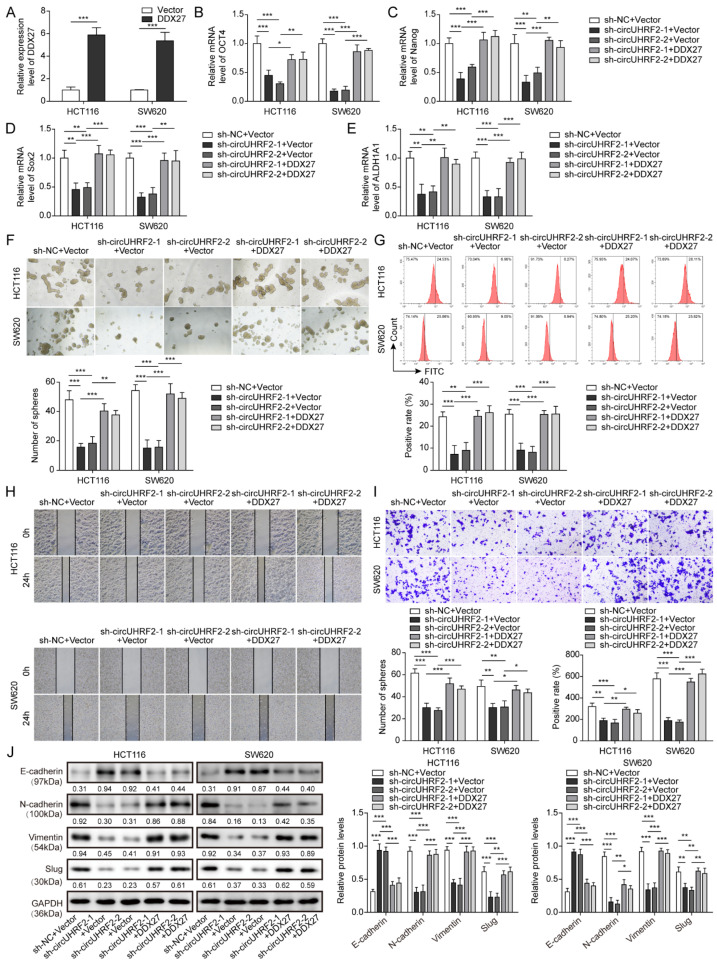
DEAD-box helicase 27 (DDX27) protein participated in circUHRF2-mediated colorectal cancer (CRC) stemness, migration, and epithelial-mesenchymal transition (EMT). HCT116 and SW480 cells were transfected with sh*circUHRF2* in the presence with or without DDX27 overexpression plasmid. The overexpression efficiency of *DDX27* mRNA was evaluated by quantitative Real-Time PCR (qRT-PCR) (**A**). Relative expression of the stemness markers, including octamer-binding transcription factor 4 (OCT4) (**B**), Nanog (**C**), SRY-box transcription factor 2 (Sox2) (**D**), and aldehyde dehydrogenase 1 family member A1 (ALDH1A1) (**E**), was assessed by qRT-PCR. (**F**) CRC sphere-forming ability was detected. (**G**) Flow cytometry analysis of CD133 expression. (**H**) A Wound-healing experiment was performed to evaluate migration ability. (**I**) Transwell assay determined invasive capacity. (**J**) Protein expression of E-cadherin, N-cadherin, Vimentin, and Slug was assessed by Western blotting, original blot see Supplementary File S1. * *p* < 0.05, ** *p* < 0.01, and *** *p* < 0.001.

To investigate the involvement of DDX27 protein in *circUHRF2*-mediated CRC development, sh*circUHRF2* with or without DDX27 overexpression plasmid was transfected into CRC cells. The overexpression efficiency of *DDX27* mRNA was confirmed by qRT-PCR (Figure 7A). Additionally, the expression of stemness markers, including *OCT4* (Figure 7B), *Nanog* (Figure 7C), *Sox2* (Figure 7D and *ALDH1A1* (Figure 7E), was markedly reduced in *circUHRF2*-silenced cells. However, DDX27 overexpression enhanced the mRNA expression of the above molecules (Figure 7B–E). In addition, *circUHRF2* depletion significantly suppressed CRC cell sphere formation efficiency (Figure 7F) and reduced the number of CD133-positive cells (Figure 7G), whereas the inhibitory effect was diminished in the cells co-transfected with DDX27 overexpressing vector (Figure 7F,G). As shown in Figure 7H,I, silencing of *circUHRF2* repressed CRC cell migration and invasion ability, which was abolished by DDX27 overexpression. Moreover, overexpression of DDX27 also counteracted sh*circUHRF2*-mediated inhibition in EMT progression (Figure 7J). In Addition, CRC cells were transfected with shDDX27 together with or without *circUHRF2* overexpression plasmid. The silencing efficiency of *DDX27* mRNA and protein was confirmed by qRT-PCR and Western blotting (Supplementary Figure S2A,B). Accordingly, DDX27 depletion restrained the expression of *OCT4, Nanog, Sox2, ALDH1A1*, CRC cell migration, invasion, and EMT, reduced sphere formation efficiency and the number of CD133-positive cells; however, these changes could be counteracted by *circUHRF2* overexpression (Supplementary Figure S2C–K). Collectively, *circUHRF2* inhibition delayed the malignant development of CRC cells via regulating the DDX27 protein.

### 3.8. Knockdown of circUHRF2 or METTL3 Suppressed CRC Growth, Stemness, and Metastasis in Nude Mice through Regulation of DDX27 Protein

**Figure 8:**
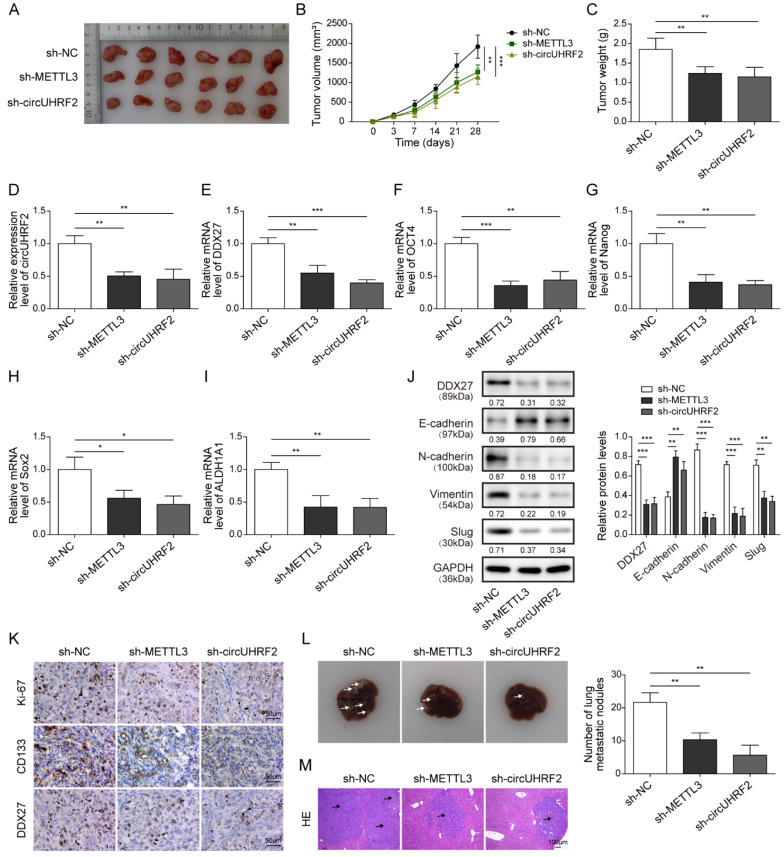
Knockdown of *circUHRF2* suppressed tumor growth, stemness, and liver metastasis in nude mice through regulation of DEAD-box helicase 27 (DDX27) protein expression. Tumor size (**A**), volume (**B**), and weight (**C**) were recorded. Quantitative Real-Time PCR (qRT-PCR) analysis of expression levels of *circUHRF2* (**D**), *DDX27* mRNA (**E**), octamer-binding transcription factor 4 (OCT4) (**F**), Nanog (**G**), SRY-box transcription factor 2 (Sox2) (**H**), and aldehyde dehydrogenase 1 family member A1 (ALDH1A1) (**I**) in tumors. (**J**) The protein expression of DDX27, E-cadherin, N-cadherin, Vimentin, and Slug was detected by Western blotting, original blot see Supplementary File S1. (**K**) Immunohistochemistry analysis of the expression of DDX27 protein, CD133, and Ki-67 in tumor sections. (**L**) Liver metastasis of colorectal cancer (CRC) was observed. (**M**) The metastatic nodules in livers were assessed by Hematoxylin and Eosin (H&E) staining. Arrows indicated the metastatic nodules. * *p* < 0.05, ** *p* < 0.01, and *** *p* < 0.001.

Finally, we validated the obtained cellular results in nude mice in vivo. Silencing of METTL3 or *circUHRF2* effectively delayed tumor growth and resulted in smaller (Figure 8A,B) and lighter tumors (Figure 8C). Knockdown of METTL3 or *circUHRF2* suppressed *circUHRF2* expression in tumor tissues (Figure 8D). Moreover, qRT-PCR analysis showed that expression of *DDX27* mRNA (Figure 8E) and stemness markers, including *OCT4* (Figure 8F), *Nanog* (Figure 8G), *Sox2* (Figure 8H), and *ALDH1A1* (Figure 8I), were significantly inhibited by METTL3 or *circUHRF2* depletion. As assessed by Western blotting, inhibition of METTL3 or *circUHRF2* strikingly decreased the expression of DDX27 protein, N-cadherin, Vimentin, and Slug, while increasing E-cadherin expression (Figure 8J). Notably, the IHC image illustrated that protein expression of Ki-67, CD133, and DDX27 was evidently inhibited in the shMETTL3 or sh*circUHRF2* group (Figure 8K). To examine the effect of circUHRF1 and METTL3 on hepatic metastasis, we collected liver tissues from the indicated groups and observed fewer metastasis nodules after the downregulation of METTL3 or *circUHRF2* (Figure 8L). Moreover, H&E staining substantiated that silencing of *circUHRF2* or METTL3 effectively ameliorated liver metastases of CRC (Figure 8M). To sum up, METTL3 or *circUHRF2* inhibition repressed CRC proliferation, stemness, and liver metastasis in vivo via suppressing DDX27 protein expression.

## 4. Discussion

CRC is a highly malignant tumor occurred in the colon or rectum. The mortality rate has been appreciably decreasing in the past 30 years, largely due to the improvement in cancer management and screenings [23]. However, for most patients with distant metastasis, the 5-year survival rate was estimated to be as low as 14% [2]. A comprehensive understanding of the pathological mechanisms of CRC would be extremely helpful for the development of life-saving therapies. In the present work, we demonstrated that m^6^A-modified *circUHRF2* by METTL3 contributed to CRC stemness and metastasis by recruiting IGF2BP1 to suppress the loss of DDX27 protein, highlighting the potential of *circUHRF2* intervention as a therapeutic strategy for CRC.

CircRNAs constitute a distinct class of non-coding RNAs with unique structures and fundamental cellular functions. Aberrant expression of circRNAs has been observed in different cancer types, and they affect cancer pathogenesis by acting as sponges or decoys for miRNA or protein [24]. Zhou et al. documented that *circ_0001666* restrained EMT and stemness of CRC cells via modulating miR-576-5p/protocadherin 10 (PCDH10) pathway [25]. A recent study demonstrated that *circ_0026628* contributed to CRC cell stemness and metastasis through elevating Sp1 transcription factor (SP1) expression to promote Wnt/β-catenin pathway activation [26]. UHRF2 functioned as a positive or negative regulator in various cancers, including CRC [27]. However, whether and how its circRNA isoform is involved in CRC carcinogenesis remains unclear. Our work provided first in vitro and in vivo evidence that *circUHRF2* knockdown efficiently repressed CRC stemness, migration, and EMT properties.

As the most prevalent posttranslational modification on eukaryotic RNAs, m^6^A profoundly regulates RNA expression during cancer progression. Elevated or declined expression of the essential m^6^A-catalyst METTL3 was reported in diverse cancer types [28]. Notably, it has been shown that METTL3 participated in CRC pathogenesis via activation of the m^6^A-glucose transporter 1 (GLUT1)-mTORC1 pathway [29]. METTL3 exerted oncogenic roles in CRC by enhancing SOX2 expression in an m^6^A-IGF2BP2-dependent manner [14]. A previous study reported that METTL3-mediated m^6^A modification of ankyrin repeat and LEM domain containing 1 (ANKLE1) acted as a cancer regulator mediated CRC cell growth and maintained genomic stability [30]. However, the implication of METTL3- m^6^A modification of *circUHRF2* in CRC tumorigenesis and metastasis is still obscure. In accordance with the previous findings, we validated that METTL3 promoted CRC cell metastasis and stemness via raising *circUHRF2* expression by m^6^A modification.

M^6^A-reader IGF2BP1 is a potent oncogene that regulates intracellular communication by stabilizing target mRNAs. For example, IGF2BP1 facilitated hthe stemness of liver cancer cells by enhancing mannoside acetylglucosaminyltransferase 5 (*MGAT5*) mRNA stability via m^6^A modification [31]. Zhang et al. documented that upregulation of IGF2BP1 promoted endometrial cancer development via m^6^A-mediated stabilization of paternally expressed 10 (PEG10) [32]. IGF2BP1, as an m^6^A reader, was demonstrated to play oncogenic roles via increasing *c-Myc* mRNA stability and level during CRC tumorigenesis [33]. Moreover, increased expression of IGF2BP1 in CRC patients contributed to the disease aggressiveness by promoting the colony-formation capacity [15]. Notably, a previous study revealed that circXPO1 binds with IGF2BP1 to raise catenin beta 1 (*CTNNB1*) mRNA stability, thereby promoting lung adenocarcinoma progression [34]. However, the interaction between circRNAs and IGF2BP1 in CRC has not been clarified. In line with previous observations, this study identified that *circUHRF2* and IGF2BP1 formed a molecular complex in the cytoplasm of CRC cells, suggesting the functional interplay between them.

DDX27 is a member of the RNA helicase family and is highly expressed in several cancers, including breast cancer [35] and CRC [36]. A previous observation found that upregulation of DDX27 exerted oncogenic function via increasing stem cell-like activity in CRC [37]. Tang et al. suggested that DDX27 overexpression was responsible for the growth and metastasis of CRC cells via activation of the NF-κB pathway [36]. However, the upstream regulatory mechanism of DDX27 in CRC is not understood. In this work, we first demonstrated that *circUHRF2* restrained the loss of DDX27 protein via the recruitment of IGF2BP1 in CRC cells, thus contributing to CRC stemness and metastasis.

## 5. Conclusions

In conclusion, our data, for the first time, identified the oncogenic roles of *circUHRF2* in CRC tumorigenesis. Our work depicted the molecular mechanisms underlying CRC stemness and metastasis that METTL3 enforced *circUHRF2* expression through m^6^A modification and subsequent inhibition of loss of DDX27 protein via recruiting IGF2BP1. These findings may pave the way for the development of efficient treatment for CRC patients.

## Figures and Tables

**Table 1:** Primers used for qRT-PCR analysis.

Genes	Primer Sequences (5′-3′)
*circUHRF2*	F: 5′-TTCAGACTGTGTTGCTGCTGAT-3′
	R: 5′-CAGGAAGATGATCAGGGTCTGG-3′
*OCT4*	F: 5′-CCCGAAAGAGAAAGCGAACC-3′
	R: 5′-GCAGCCTCAAAATCCTCTCG-3′
*Nanog*	F: 5′-GTCCCAAAGGCAAACAACCC-3′
	R: 5′-ATCCCTGCGTCACACCATTG-3′
*Sox2*	F: 5′-GCCCTGCAGTACAACTCCAT-3′
	R: 5′-GACTTGACCACCGAACCCAT-3′
*ALDH1A1*	F: 5′-GATCCCCGTGGCGTACTATG-3′
	R: 5′-TGGATCTTGTCAGCCCAACC-3′
*METTL3*	F: 5′-GAGTGCATGAAAGCCAGTGA-3′
	R: 5′-CTGGAATCACCTCCGACACT-3′
*DDX27*	F: 5′-CCGCAGTGCTGATTTCAACC-3′
	R: 5′-GCTCCAGGCTGAGGAAATGG-3′
*GAPDH*	F: 5′-CCAGGTGGTCTCCTCTGA-3′
	R: 5′-GCTGTAGCCAAATCGTTGT-3′

Abbreviations: qRT-PCR, quantitative Real-Time PCR; OCT4, octamer-binding transcription factor 4; Sox2, SRY-box transcription factor 2; ALDH1A1, aldehyde dehydrogenase 1 family member A1; METTL3, Methyltransferase-like 3; DDX27, DEAD-box helicase 27; GAPDH, glyceraldehyde-3-phosphate dehydrogenase.

**Table 2:** Correlation of the expression levels of *circUHRF2* with the clinicopathological characteristics of CRC patients.

Clinical Parameters	Cases (n)	*circUHRF2* Expression		*p*-Value (* *p* < 0.05)
		High (n)	Low (n)	
**Age**				0.796
<60 years	29	15	14	
≥60 years	31	15	16	
**Gender**				0.559
Male	44	23	21	
Female	16	7	9	
**Tumor size (cm)**				0.018 *
<5	25	8	17	
≥5	35	22	13	
**TNM stage**				0.035 *
I/II	24	8	16	
III/IV	36	22	14	
**Local invasion**				0.414
T1/T2	20	9	11	
T3/T4	40	21	19	
**Differentiation**				0.069
Poor	27	17	10	
Moderate/High	33	13	20	
**Lymph node metastasis**				0.19
Yes	35	20	15	
No	25	10	15	

Abbreviations: CRC, colorectal cancer; TNM, tumor nodes metastasis.

## Data Availability

All data generated or analyzed during this study are included in this published article.

## Supplementary Materials

The following supporting information can be downloaded at: https://www.mdpi.com/article/10.3390/cancers15123148/s1, Figure S1: Potential m^6^A-modified sites in *circUHRF2* were predicted by SRAMP database; Figure S2: Overexpression of cirUHRF2 rescued the CRC stemness, migration and EMT correlated with the loss of DDX27; File S1: Original Western Blots.
