# Supplementary material for: METTL3-Modulated circUHRF2 Promotes Colorectal Cancer Stemness and Metastasis through Increasing DDX27 mRNA Stability by Recruiting IGF2BP1
Source: Cancers (Basel). 2023 Jun 11;15(12):3148. doi: 10.3390/cancers15123148 (PMC10295973; doi:10.3390/cancers15123148)
Supplement: Supplementary file 1 [file cancers-15-03148-s001.zip › Supplementary Figure legends.pdf]

Supplementary information

Supplementary Figure S1. **Overexpression of cirUHRF2 rescued the CRC stemness, migration and EMT correlated with the loss of DDX27**. HCT116 and SW480 cells were transfected with shDDX27 in the presence with or without circUHRF2 overexpression plasmid. Silencing efficiency of DDX27 was evaluated by qRT-PCR (A) as well as Western blotting (B). Relative expression of the stemness markers, including OCT4 (C), Nanog (D), Sox2 (E) and ALDH1A1 (F) was assessed by qRT-PCR. (G) CRC sphere forming ability was detected. (H) Flow cytometry analysis of CD133 expression. (I) Wound healing experiment was performed to evaluate migration ability. (J) Transwell assay determined invasive capacity. (K) Protein expression of E-cadherin, N-cadherin, Vimentin and Slug was assessed by Western blotting. *$p < 0.05$, **$p < 0.01$, and ***$p < 0.001$.

Supplementary Figure S2. Potential m6A-modified sites in circUHRF2 were predicted by SRAMP database.
