# Supplementary figures and images for: METTL3-Modulated circUHRF2 Promotes Colorectal Cancer Stemness and Metastasis through Increasing DDX27 mRNA Stability by Recruiting IGF2BP1
Source: Cancers (Basel). 2023 Jun 11;15(12):3148. doi: 10.3390/cancers15123148 (PMC10295973; doi:10.3390/cancers15123148)

Fig. 2K

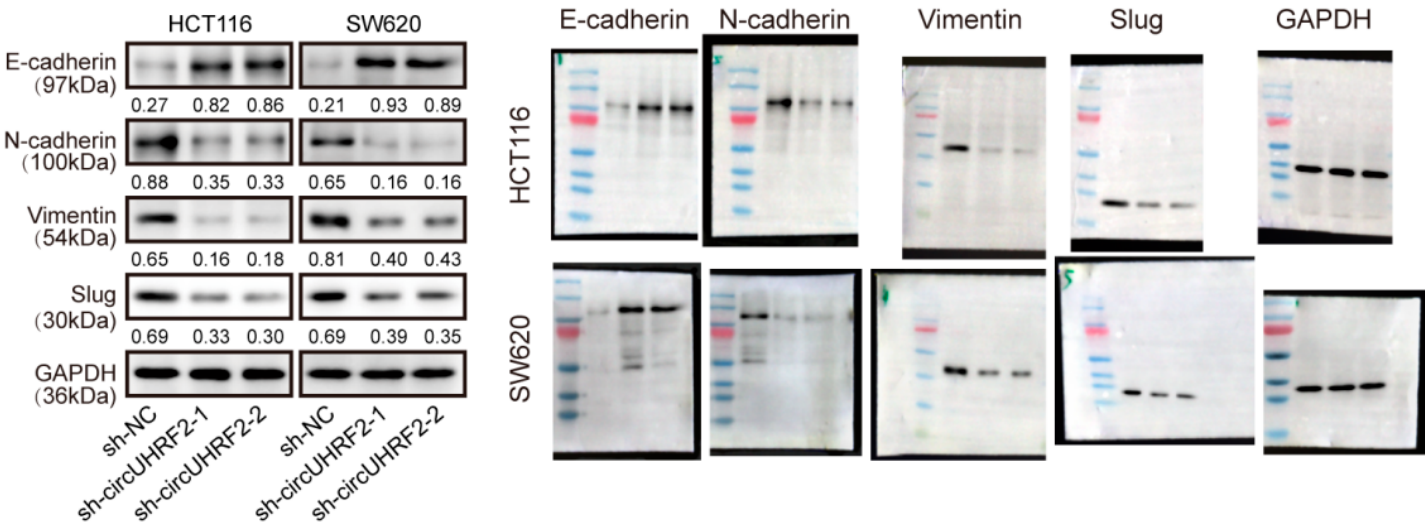

Fig. 3E

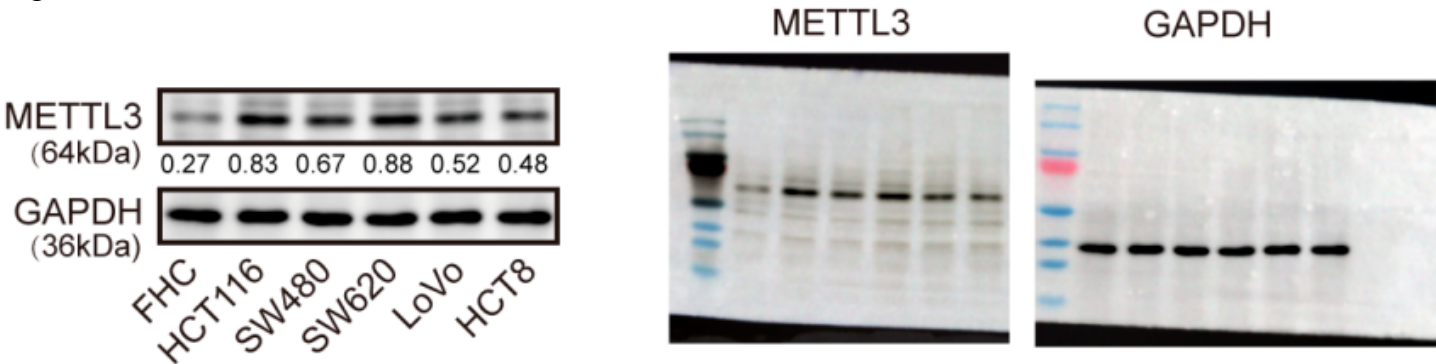

Fig. 3H

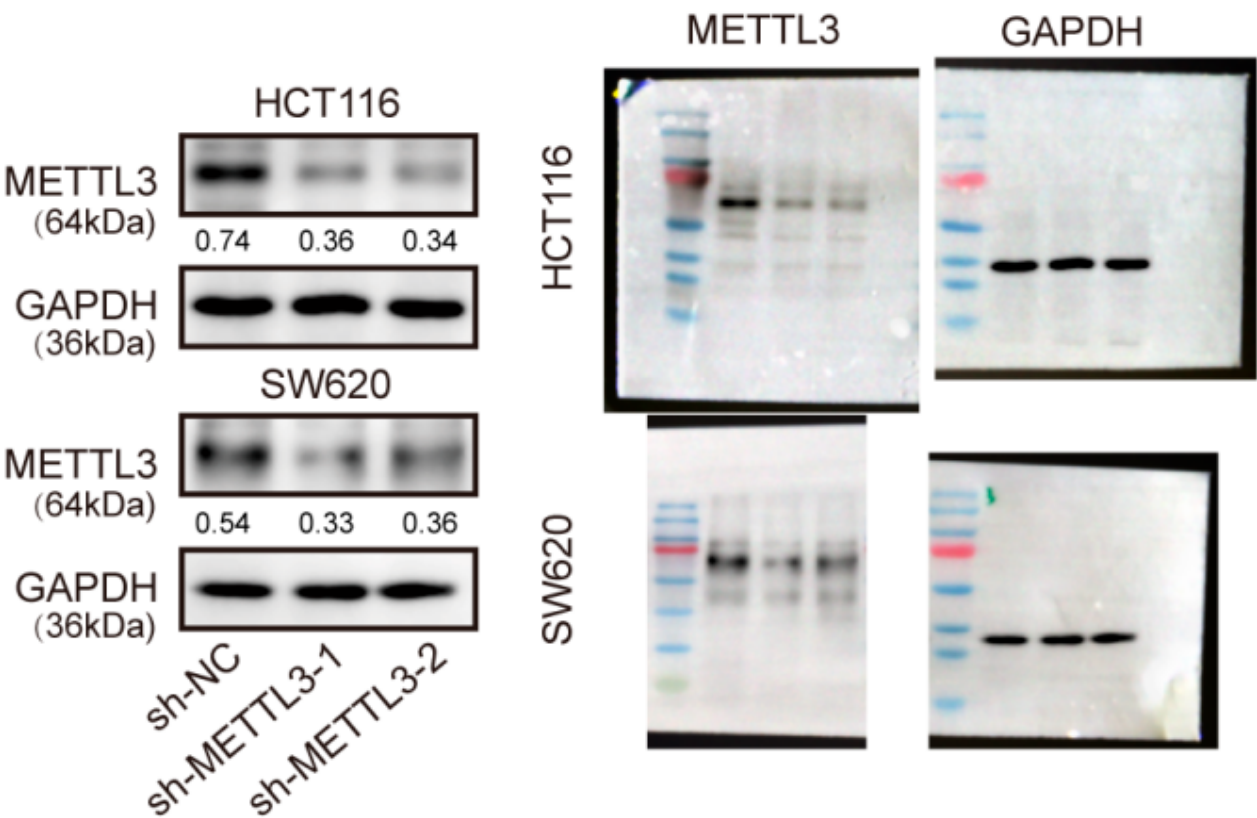

Fig. 4K

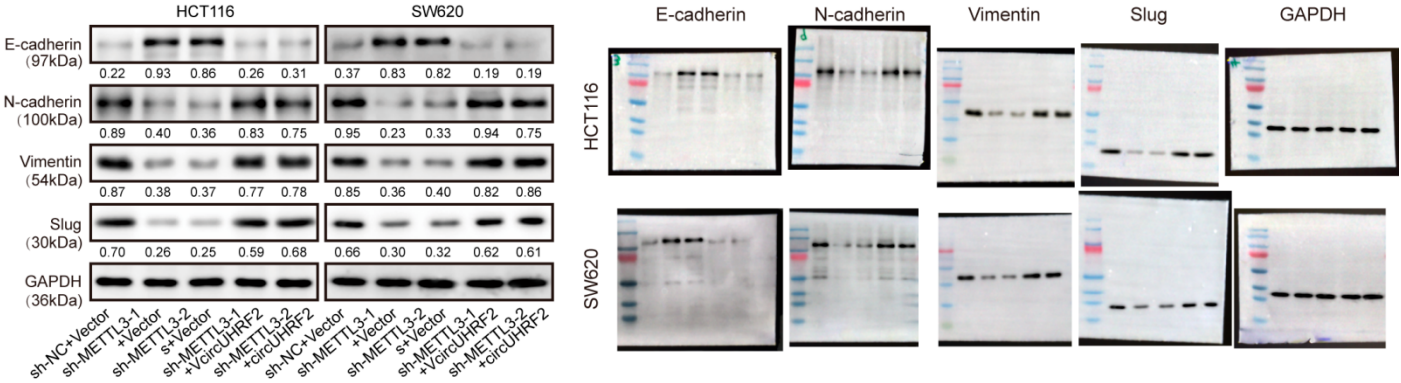

Fig. 5A

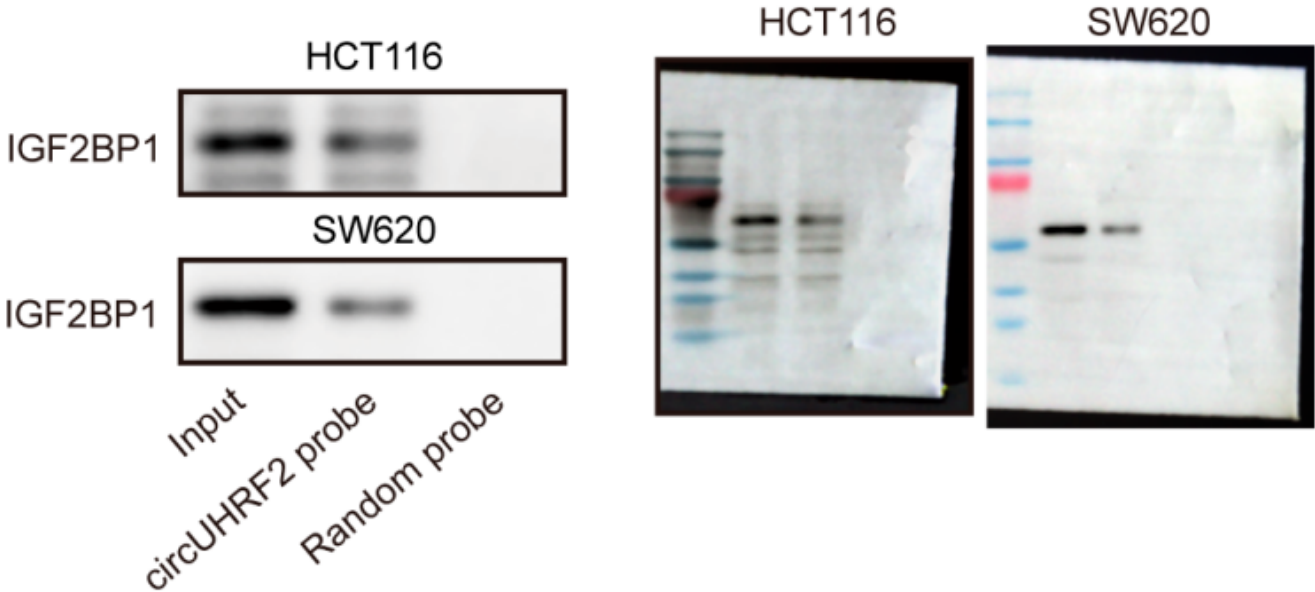

Fig. 6D

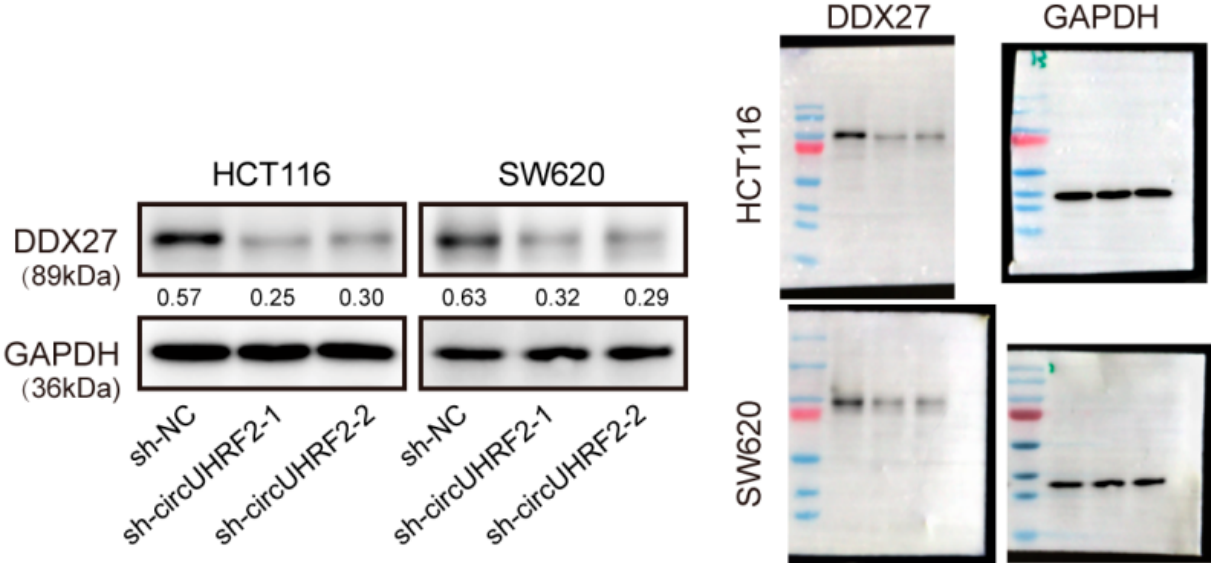

Fig. 6H

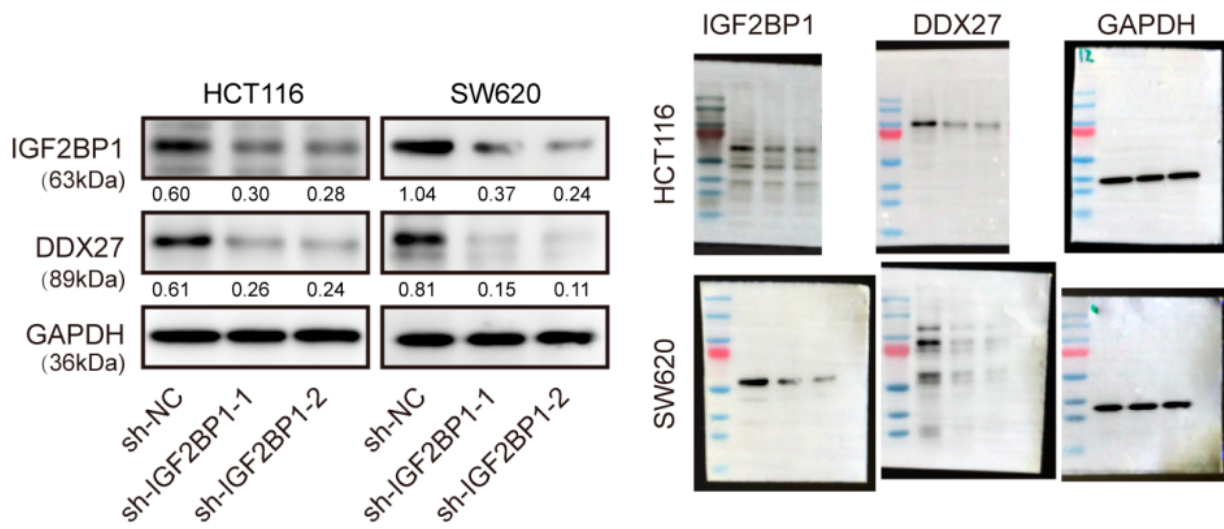

Fig. 7J

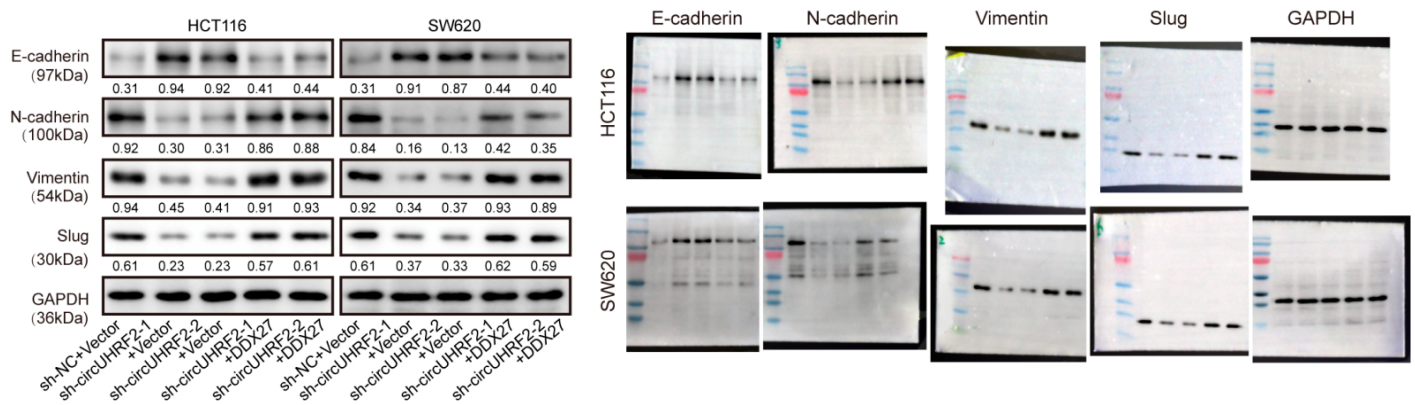

Fig. 8J

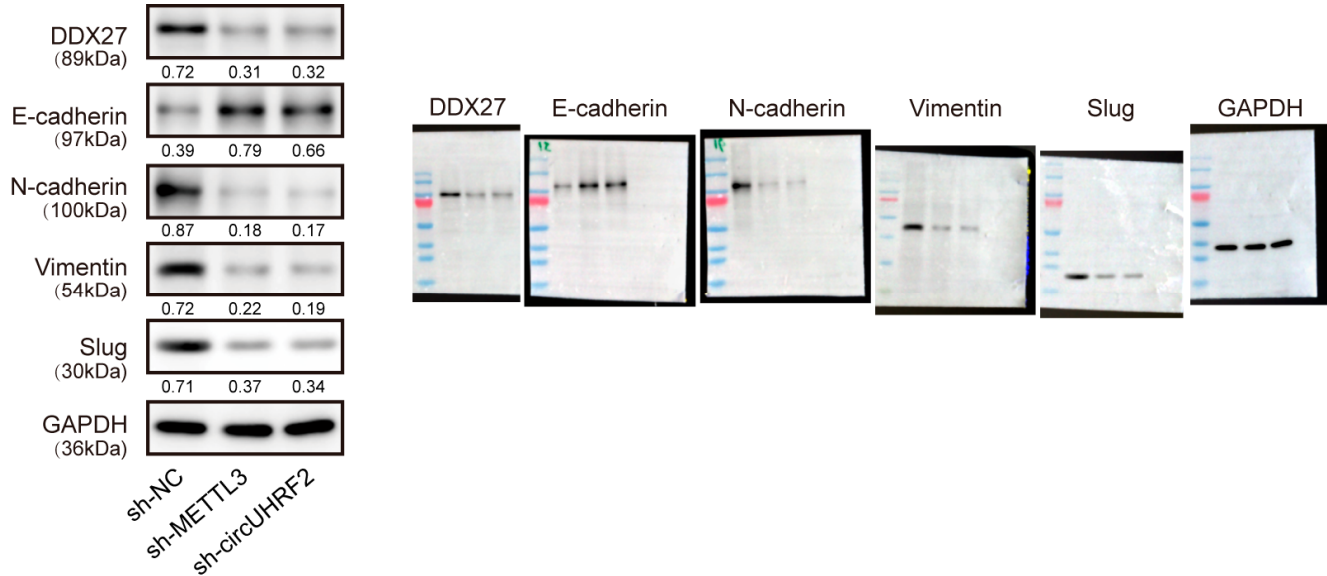

Supplementary Fig. S2K

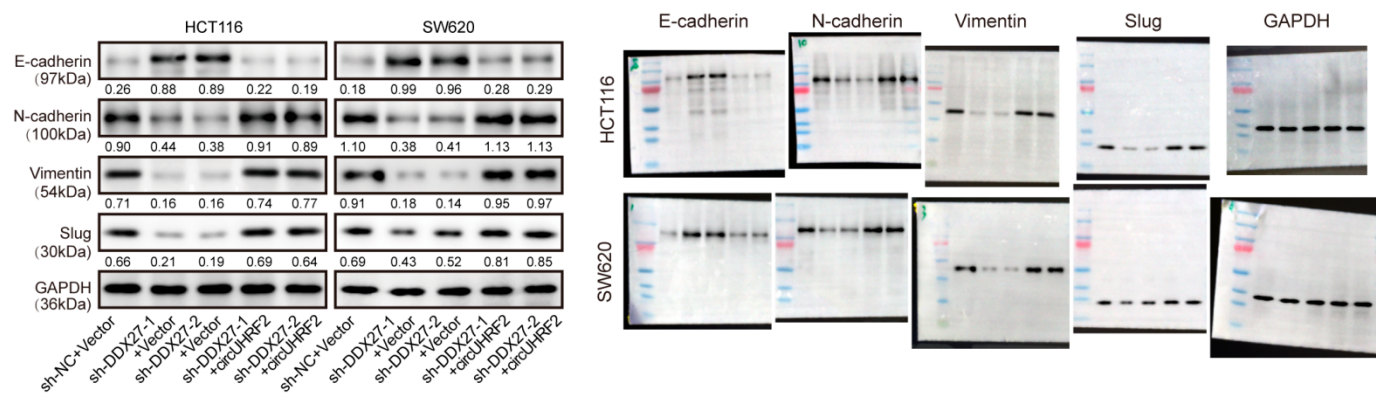

Supplement: Supplementary file 1 [file cancers-15-03148-s001.zip › File S1-Original Western Blots.pdf]

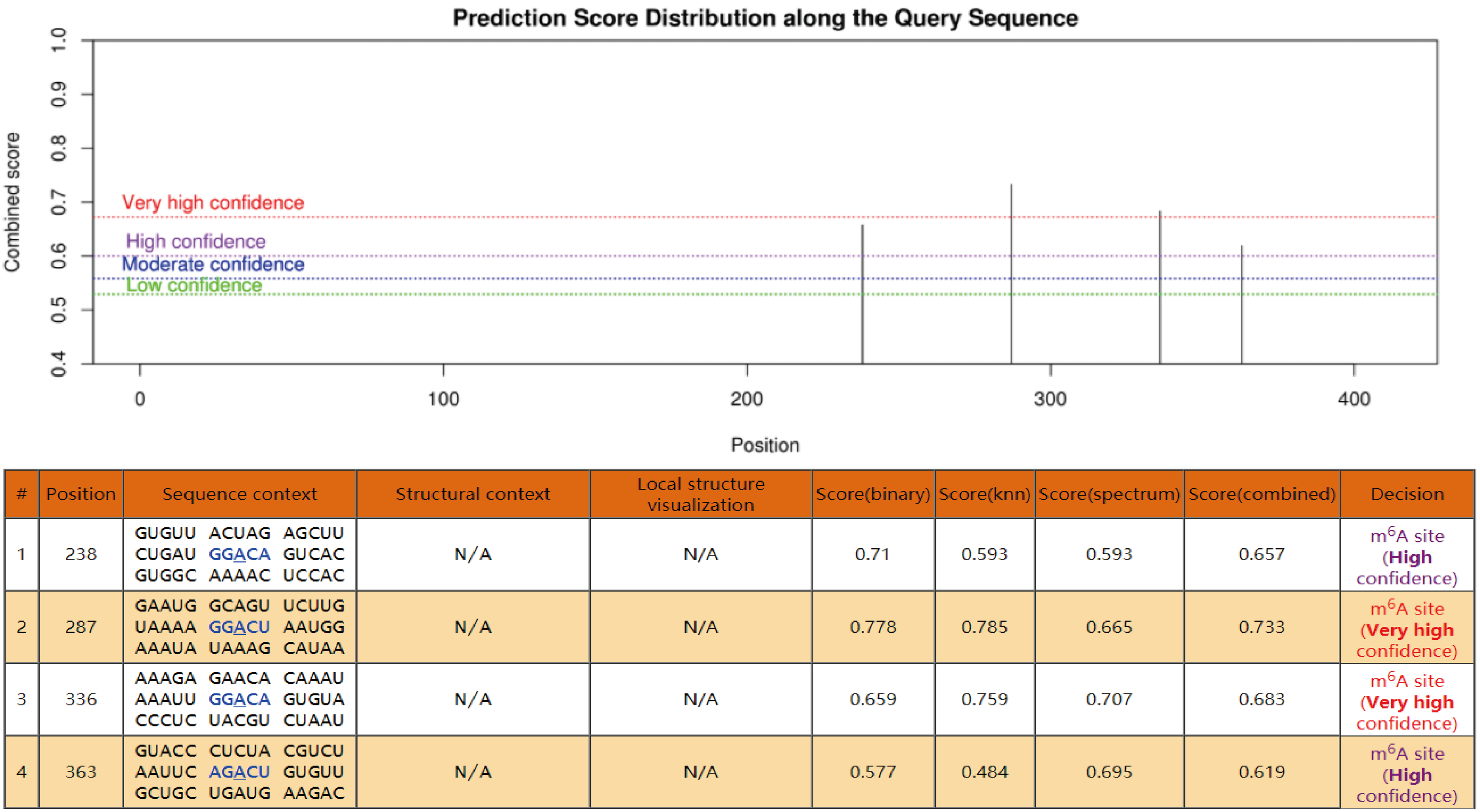

Supplement: Supplementary file 1 [file cancers-15-03148-s001.zip › Supplementary Figure S1.tif]

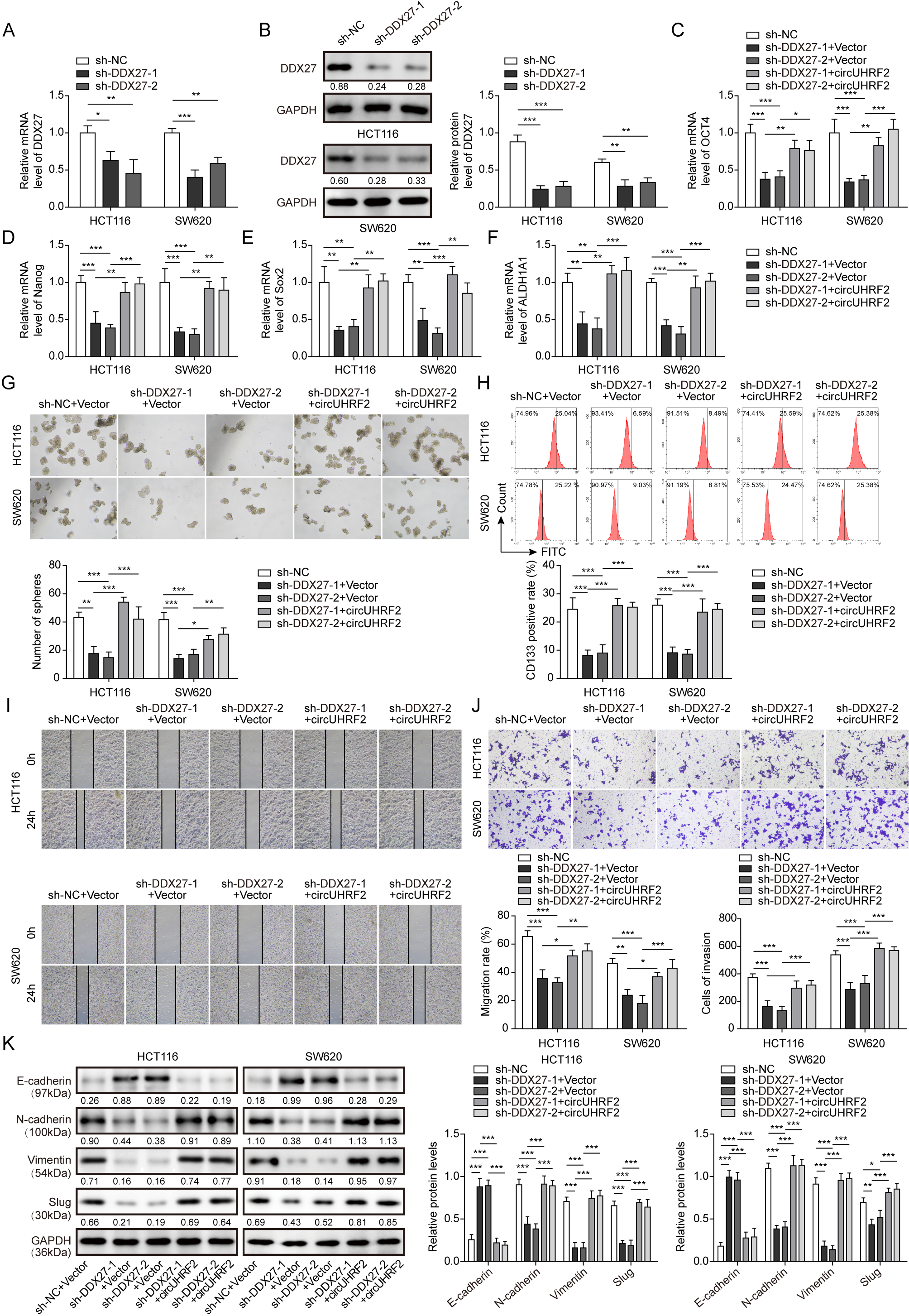

Supplement: Supplementary file 1 [file cancers-15-03148-s001.zip › Supplementary Figure S2.tif]
